# Arc/Arg3.1 has an activity-regulated interaction with PICK1 that results in altered spatial dynamics

**DOI:** 10.1038/s41598-018-32821-4

**Published:** 2018-10-02

**Authors:** Brandee M. S. S. Goo, Bethany J. Sanstrum, Diana Z. Y. Holden, Yi Yu, Nicholas G. James

**Affiliations:** Department of Cell and Molecular Biology, John A. Burns School of Medicine, 651 Ilalo St., BSB 222, University of Hawaii, Honolulu, HI 96813 USA

## Abstract

Activity-regulated cytoskeleton-associated protein (Arc; also known as Arg3.1) is an immediate early gene product that is transcribed in dendritic spines and, to date, has been best characterized as a positive regulator of AMPAR endocytosis during long-term depression (LTD) through interaction with endocytic proteins. Here, we show that protein interacting with C terminal kinase 1 (PICK1), a protein known to bind to the GluA2 subunit of AMPARs and associated with AMPAR trafficking, was pulled-down from brain homogenates and synaptosomes when using Arc as immobilized bait. Fluctuation and FLIM-FRET-Phasor analysis revealed direct interaction between these proteins when co-expressed that was increased under depolarizing conditions in live cells. At the plasma membrane, Arc-mCherry oligomerization was found to be concentration dependent. Additionally, co-expression of Arc-mCherry and EGFP-PICK1 followed by depolarizing conditions resulted in significant increases in the number and size of puncta containing both proteins. Furthermore, we identified the Arc binding region to be the first 126 amino acids of the PICK1 BAR domain. Overall, our data support a novel interaction and model where PICK1 mediates Arc regulation of AMPARs particularly under depolarizing conditions.

## Introduction

Long-term potentiation (LTP), long-term depression (LTD), and synaptic plasticity, are synaptic responses to neural stimulation that are widely thought to underlie learning and memory^1,2^. A key component in the regulation of synaptic plasticity in excitatory neurons is the trafficking of AMPA-type glutamate receptors (AMPARs)^3,4^. Changes in synaptic efficiency during LTP and LTD result in part from alterations in AMPAR endocytosis and exocytosis leading to an alteration in surface concentration of AMPARs. Regulation of surface AMPAR expression has been shown to be tightly controlled by endocytosis and exocytosis machinery, indicating that AMPARs are likely carried to and from the plasma membrane on vesicles.

One specific protein that has been highly linked to AMPAR regulation is an immediate early gene product known as Arc (Activity-regulated cytoskeleton-associated protein)^5^ or Arg 3.1 (Activity regulated gene 3.1)^6^. Arc is a ~45 kDa protein which has been shown to contribute to synaptic depression by facilitating AMPAR endocytosis on the postsynaptic membrane^7^. During LTD, Arc mRNA is transported to the postsynaptic density where translation leads to a rapid increase in the concentration of localized Arc^7,8^. AMPAR endocytosis associated with Arc has been highly characterized as a direct association with endocytic co-factors such as dynamin and endophilin^9,10^. Dynamins are ~100 kDa GTPases that catalyze membrane scission during vesiculation and have a binding region for Arc around residues 1–227^9–13^. Recent studies have further supported the role of this interaction in the postsynaptic terminal by demonstrating that Arc can stimulate the polymerization and activity of dynamin 2 and dynamin 3, while presynaptic dynamin 1 was not shown to be affected^9^. Conversely, endophilins are ~40 kDa proteins with C-terminal BAR (Bin/Amphiphysin/Rvs) domains that homodimerize into crescent-shaped structures that induce and stabilize membrane curvature at the necks of budding endocytic vesicles^9,14^. The Arc-binding determinant has been localized to residues 218–254 of endophilin which corresponds to the C-terminal of the BAR domain^10^. These results, along with the identification that Arc can self-associate into large species, demonstrate how Arc could serve as a template for recruitment of endocytosis cofactors during LTD. Despite having this endocytosis model, it is unclear how Arc specifically promotes targeted endocytosis of AMPARs. Recent evidence supports an interaction between Arc and clathrin-adaptor protein 2 (AP-2) as a molecular connection to direct Arc to AMPARs^15^.

Using biochemical and biophysical methods, we identified another protein that directly interacts with Arc that has also been reported to interact with and regulate AMPAR surface expression. Specifically, we demonstrate a novel interaction between protein interacting with C-kinase 1 (PICK1) and Arc through multiple assays. PICK1 is a BAR domain-containing protein that is implicated in the regulation of AMPAR surface expression. It also contains a PDZ (PSD95/Dlg/ZO1) domain that binds directly to AMPARs at the intracellular C-terminal domains of the GluA2 and GluA3 subunits following calcium binding^16–19^. Although some studies have linked Arc and PICK1 to dendritic spine maturation and LTP, the majority of reports indicate that both proteins promote reduction in the surface density of AMPARs in the synapse leading to LTD^8,20^. We show direct interaction between Arc and PICK1 via pull-down assays from brain lysates and synaptosomes. This interaction was also noted when using purified recombinant forms of Arc and PICK1 suggesting a direct interaction between these proteins. Using live cell fluorescence imaging we demonstrated that the Arc and PICK1 interaction occurs in the cytosol and at the plasma membrane. Quantitative fluctuation analysis revealed that 10% of total Arc protein was found in complex with PICK1. Interestingly, this interaction was increased when transfected cells were analyzed under depolarizing conditions. Finally, we show that Arc likely interacts directly with the BAR domain of PICK1. These findings are the first to show a direct interaction between Arc and PICK1 as well as an enhanced association following depolarization. Overall, we propose that PICK1 initially binds to AMPARs and facilitates an interaction with Arc to recruit endocytic cofactors to selectively reduce AMPAR expression and initiate LTD.

## Results

### Arc directly interacts with PICK1 *in vitro*

Arc functions to regulate postsynaptic transmission through facilitation of AMPAR endocytosis, which is proposed to occur by direct coordination with the endocytic machinery^9,10,15^. However, the mechanism of how Arc selects AMPARs as cargo has yet to be fully addressed. It is likely that Arc targets these receptors through an interaction with a protein that has direct binding to both AMPARs and Arc. Therefore, we sought to identify potential binding partners of Arc that are also known to associate with AMPARs by conducting a pull-down with GST-Arc and whole mouse brain lysates using purified GST protein as a negative control. Brain lysates were incubated with GST or GST-Arc, which was then bound to glutathione resin. Using Westernblot (WB) we screened proteins that have known interactions with AMPARs and identified PICK1 as a potential direct interacting protein of Arc (Supp. Fig. 1). This finding was of particular interest because Arc and PICK1 have been linked to AMPAR surface expression^7,8,21^.

**Figure 1:**
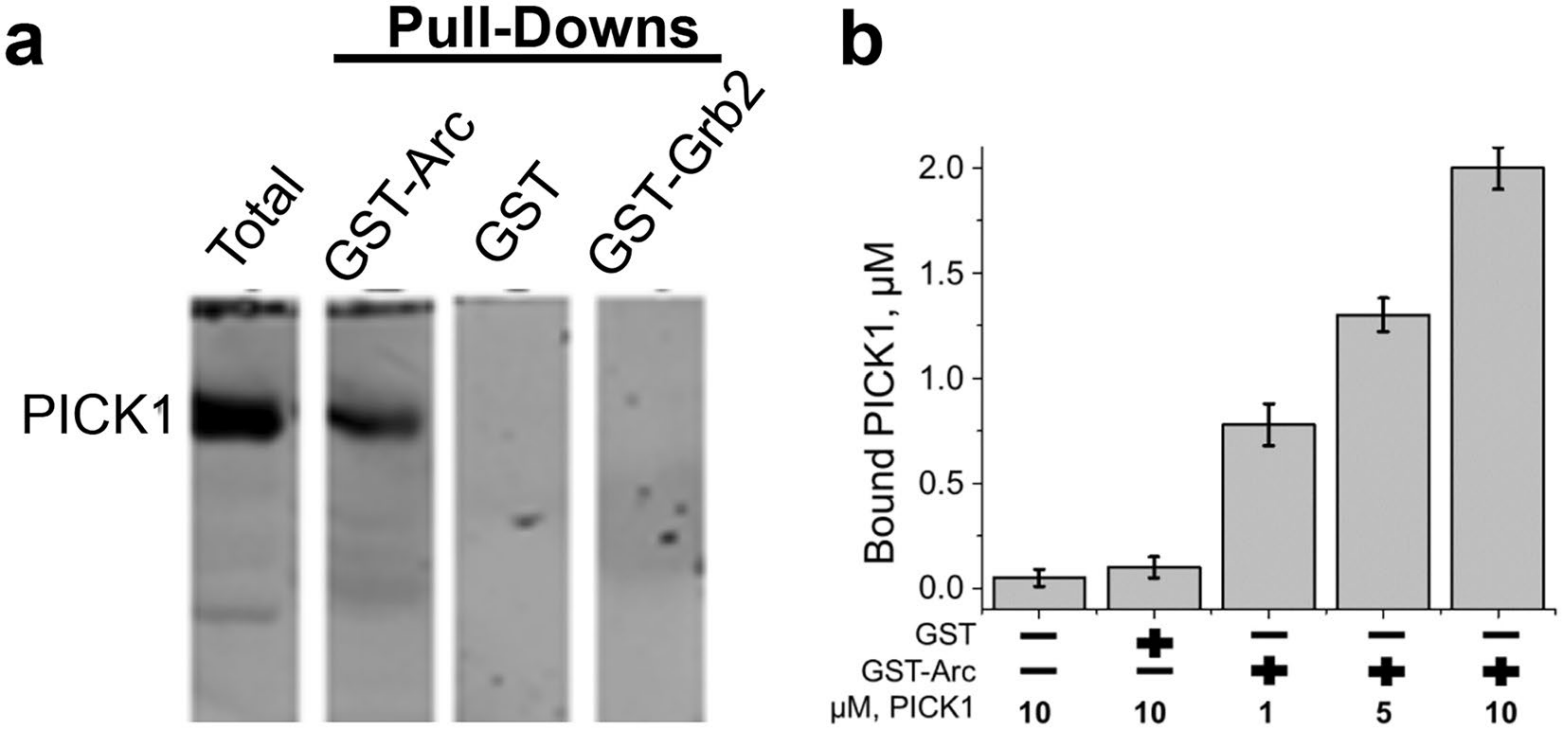
GST-Arc precipitates with PICK1 from synaptosome lysates and purified protein. (**a**) Westernblot (WB) analysis of pull-downs from synaptosome lysates using glutathione resin shows an association between Arc and PICK1. Lane 1 shows the entire synaptosome lysate as a positive control. Lane 2 depicts the GST-Arc pull-down and is positive when blotting for PICK1. Lane 3 and 4 were incubated with GST or GST-Grb2 respectively and were used as negative controls. A fixed volume of GST, GST-Arc, and GST-Grb2 at 10 µM was used throughout each condition. (**b**) Glutathione resin bound to purified GST or GST-Arc was used to show a direct interaction with purified His-PICK1 and GST-Arc. Bound PICK1 was calculated as the concentration of His-PICK1 free in solution post incubation subtracted from the total concentration of His-PICK1 added to the resin. Error bars depict the standard deviation over all trials.

To determine if this interaction occurs at the membrane of brain lysates where AMPAR regulation is known to occur, synaptosome fractions were generated and analyzed using GST-Arc pull-downs (as discussed in the methods section). A large fraction of the total PICK1 was successfully pulled-down with GST-Arc in these samples but was not found in samples incubated with GST or GST-Grb2 control proteins (Fig. 1a, Supp. Fig. 2). We then expressed and purified recombinant His-PICK1 to demonstrate a direct interaction with GST-Arc bound to glutathione resin and eliminate the possibility that other proteins are mediating this interaction. Quantitative analysis of bound PICK1 demonstrated that there was an increase in captured protein when incubated with beads containing GST-Arc compared to GST or beads alone (Fig. 1b). This suggests that PICK1 does directly bind with Arc and that this interaction can be noted in the membrane fraction of brain lysates indicating that it could be involved in the regulation of AMPAR endocytosis.

### Interaction between Arc and PICK1 occurs in live cells

**Figure 2:**
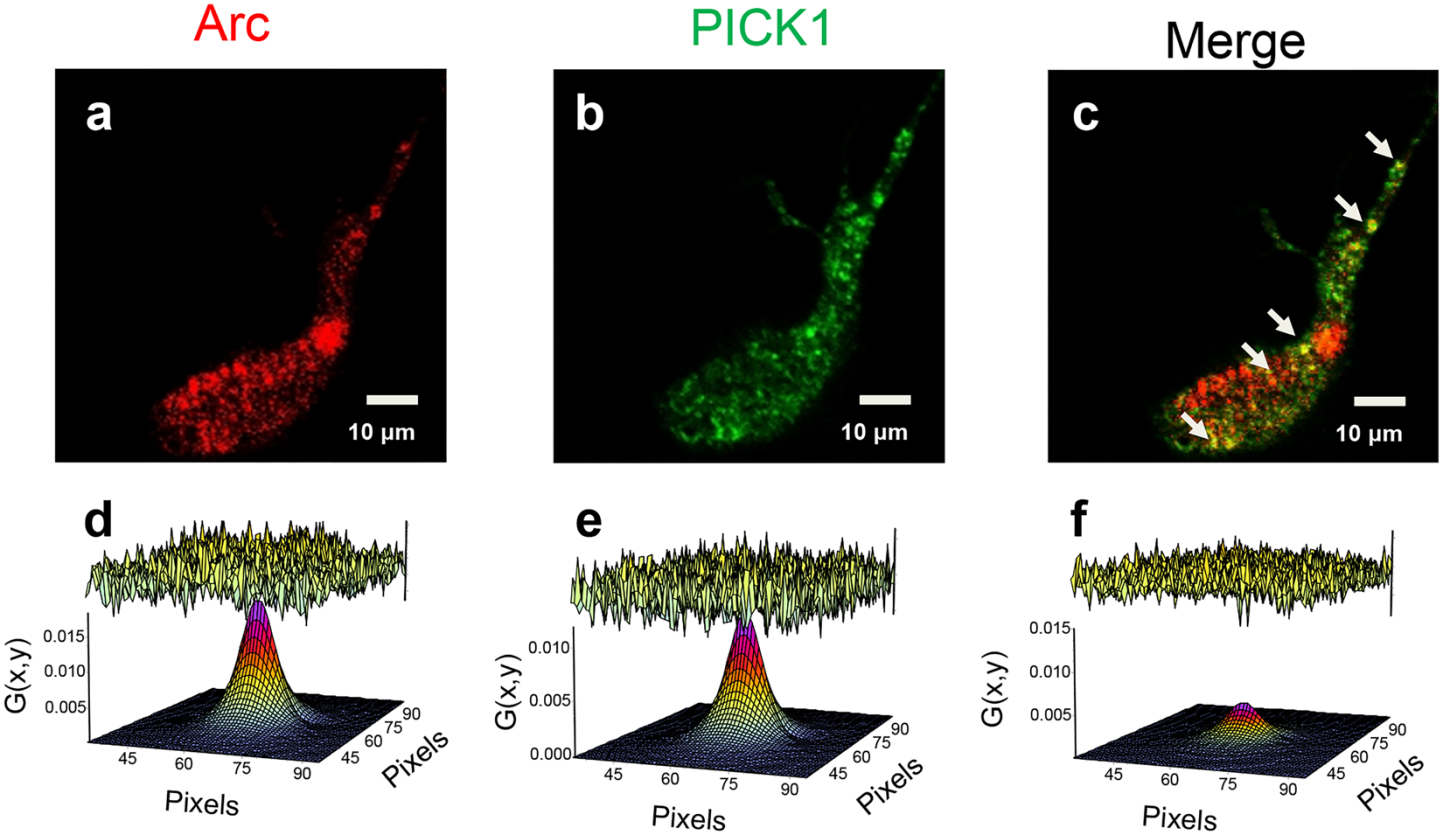
Arc and PICK1 form complexes in live cells. **(a–c**) Intensity images of a representative SH-SY5Y cell co-transfected with Arc-mCherry and EGFP-PICK1 show co-localization in puncta. (**d–f**) ccRICS analysis demonstrates directed interaction of Arc-mCherry and EGFP-PICK1 in live cells. Cross-correlation of the fluctuation from Arc and PICK1 (**d,e**) produced a new correlation curve (**f**) of only the fluorophores which are moving together in the cells. The curve is smaller because the correlated signal is only a fraction of the total particles in each channel. Diffusion obtained in the correlated sample is similar to that obtained from individual RICS analysis. Lack of interaction would be predicted to produce a randomized, non-correlated signal which was observed for our control conditions.

It is important to establish that this interaction is also present in a live-cell system and can occur during normal cellular functions. To do this we used fluorescence fluctuation analysis of transfected SH-SY5Y cells, which provided further support of a direct interaction between these two proteins under more physiologically relevant conditions. (For an overview of the fluorescence methodology used we recommend the review by Jameson, Ross, and Albanesi)^22^. Prior to experimentation, we established that no interaction occurs between our fluorescent constructs and the opposing fluorescent tag (Arc-mCherry with EGFP and EGFP-PICK1 with mCherry). Both Arc-mCherry and EGFP-PICK1 have fairly dispersed cytosolic distributions that form distinct puncta throughout the cell (Fig. 2). Merged intensity images show areas of co-localization between Arc-mCherry and EGFP-PICK1 expression (Fig. 2a–c). Changes in cytosolic oligomerization were quantified using a photon-counting histogram (PCH) model to compare the changes in the self-association of Arc-mCherry and EGFP-PICK1 to monomeric fluorescent protein standards in co-transfected cells. These findings are quantified in Table 1 as average and normalized brightness (ε). Interestingly, co-transfection of both Arc-mCherry and EGFP-PICK1 showed significant changes in Arc-mCherry oligomerization (normalized ε from 1.11–1.37) but did not have a significant effect on EGFP-PICK1 (normalized ε from 1.38–1.40) when compared to co-transfection with monomeric standards (Table 1). This finding suggests that while the presence of PICK1 affects Arc self-association in the cytosol of live cells, Arc has little effect on the oligomerization of PICK1.

**Table 1:** Fluctuation analysis of cytosolic Arc-mCherry and EGFP-PICK1.

Transfected Protein (s)^a^	Ave. Diffusion (µm^2^/sec)^b^	Ave. ε (cpsm)	Norm. ε^d^
EGFP	9.0 ± 3.0	4,500 ± 500	1.00
EGFP-PICK1 + mCherry	1.0 ± 0.9	6,200 ± 1,200	1.38
EGFP-PICK1 + Arc-mCherry	1.4 ± 0.7	6,300 ± 1,100	1.40
mCherry	9.1 ± 2.5	3,500 ± 500	1.00
Arc-mCherry + EGFP	1.9 ± 0.9	3,900 ± 1,100	1.11
Arc-mCherry + EGFP-PICK1	1.1 ± 0.7	4,800 ± 1,500^c^	1.37^c^

^a^For each condition, n > 20 with concentrations of transfected proteins ranging from 20 to 1,500 nM. No significant trend was found for concentration and thus each concentration was included in the final calculations.

^b^p < 0.05 from a multivariate ANOVA using Bonferroni post-hoc when control samples, EGFP and mCherry, where compared to the experimental conditions.

^c^p < 0.05 from a multivariate ANOVA using Bonferroni post-hoc when control sample, mCherry, was compared to Arc-mCherry + EGFP-PICK1.

^d^Values were generated by dividing by monomer controls to each corresponding sample.

Since we were able to determine oligomerization differences between Arc-mCherry and EGFP-PICK1, we next wanted to determine if there were any alterations to the cytosolic diffusion due to co-transfection. Raster Imaging Correlation Spectroscopy (RICS) analysis showed that the diffusion of both Arc-mCherry and EGFP-PICK1 was not affected by co-transfection but was significantly slower than mCherry and EGFP controls suggesting that these constructs are interacting with slow moving cellular components (Table 1). Baseline RICS 3-D autocorrelation function (ACF) curves of Arc-mCherry and EGFP-PICK1 produced autocorrelation functions with a G(0) value around 0.02 and 0.015 respectively (Fig. 2d,e). Next, we used cross-correlation RICS (ccRICS) in which the signal from the red channel is correlated with signal from the green channel, to determine if Arc-mCherry and EGFP-PICK1 are associated. The presence of a correlation using ccRICS indicates that the two proteins are moving together within the cell. A ccACF curve was obtained between Arc-mCherry and EGFP-PICK1 with a G(0) = 0.005 (Fig. 2f). Comparison of the G(0) values indicates that ~10% of the Arc-mCherry is associated with EGFP-PICK1 in a complex within the cytosol confirming the co-localization results and providing strong evidence that Arc and PICK1 also interact in a live cell system.

### Activity induced interaction leads to redistribution of PICK1 with Arc on the plasma membrane

**Figure 3:**
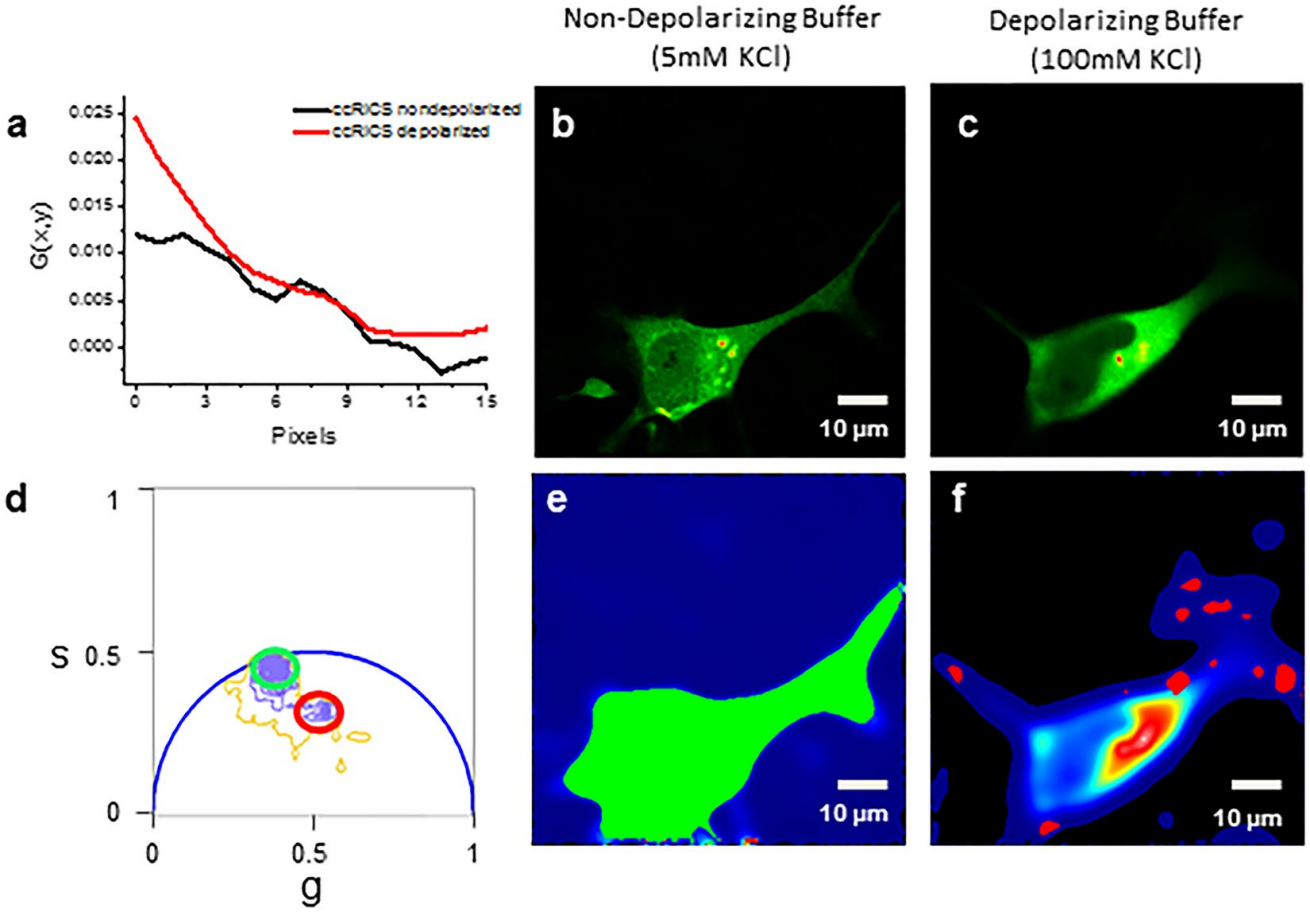
Depolarization of SH-SY5Y cells increases the interaction between Arc-mCherry and EGFP-PICK1. (**a**) The amount of Arc/PICK1 complex formation is increased under depolarizing conditions based on the G(0) values from the cross-correlation functions obtained from RICS (ccRICS) analysis. (**b,c**) Intensity images of EGFP-PICK1 in non-depolarizing and depolarizing buffer (respectively) show areas of PICK1 localization. (**d**) Phasor points of a co-transfected cell under depolarizing conditions show a population of unquenched pixels (no interaction) circled in green with a lifetime of 2.3 ns and a distinct second population of quenched pixels (interaction between Arc-mCherry and EGFP-PICK1) circled in red with a much lower lifetime of 1.8 ns. (**e,f**) Spatial arrangement of phasor points on representative non-depolarized and depolarized cells (respectively) indicate the location of pixels that do not show FRET (**e**; green) and pixels showing FRET (**f**; red).

Arc is an immediate early gene that is rapidly translated at the site of depolarization following neural activity^23^. Therefore, in order to determine if the Arc/PICK1 interaction is increased under depolarizing conditions, we incubated co-transfected cells in non-depolarizing buffer (5 mM KCl) or depolarizing buffer (100 mM KCl) for 15 minutes prior to data collection. Depolarization resulted in increased Arc-mCherry and EGFP-PICK1 complex formation (~20% ± 4% S.D.) as determined by an increased G(0) value (Fig. 3a). This suggests that Arc interaction with PICK1 may be increased by depolarization. In order to further quantify changes in interaction due to depolarization we utilized fluorescence lifetime imaging microscopy (FLIM) to detect Förster resonance energy transfer (FRET) with phasor plot analysis (Fig. 3b–f). In a phasor plot representation, the lifetime of each pixel is plotted and movement into the universal circle indicates a decrease in lifetime and therefore is associated with energy transfer and suggests the presence of protein-protein interaction. (Further explanation of the method may be obtained in overview by Digman and colleagues)^24^. During co-transfection with Arc-mCherry, the phasor points of EGFP-PICK1 were shifted towards a second shorter lifetime (red circle) that indicates areas of direct protein-protein interaction (Fig. 3b–f; Table 2). While 10% of cells transfected with EGFP-PICK1 alone showed a second lifetime due to quenching, this was slightly enhanced when co-transfected with Arc-mCherry and greatly enhanced when under depolarizing conditions. These results support our *in vitro* data in establishing a direct Arc/PICK1 interaction that is enhanced by depolarization.

**Table 2:** Average fluorescence lifetimes of EGFP-PICK1 in depolarized and non-depolarized cells.

Transfected Protein (s)^a^	Depolarized	Ave. Lifetime 1 (ns)	Ave. Lifetime 2 (ns)	% showing FRET	FRET efficiency
EGFP	N	2.41 ± 0.03	—	—	—
EGFP	Y	2.43 ± 0.01	—	—	—
EGFP-PICK1	Y	2.40 ± 0.03	1.93 ± 0.05	10%	10% (±6%)
EGFP-PICK1 + Arc-mCherry	N	2.38 ± 0.04	1.95 ± 0.01	15%	18% (±9%)
EGFP-PICK1 + Arc-mCherry	Y	2.37 ± 0.05	2.00 ± 0.20	60%	28% (±10%)

^a^For each condition, n > 20.

The average lifetime and standard deviation are presented for each group. If two lifetimes were present based on the phasor plot distribution a second average lifetime and standard deviation are presented along with the percentage of cells showing FRET and the FRET efficiency observed.

**Figure 4:**
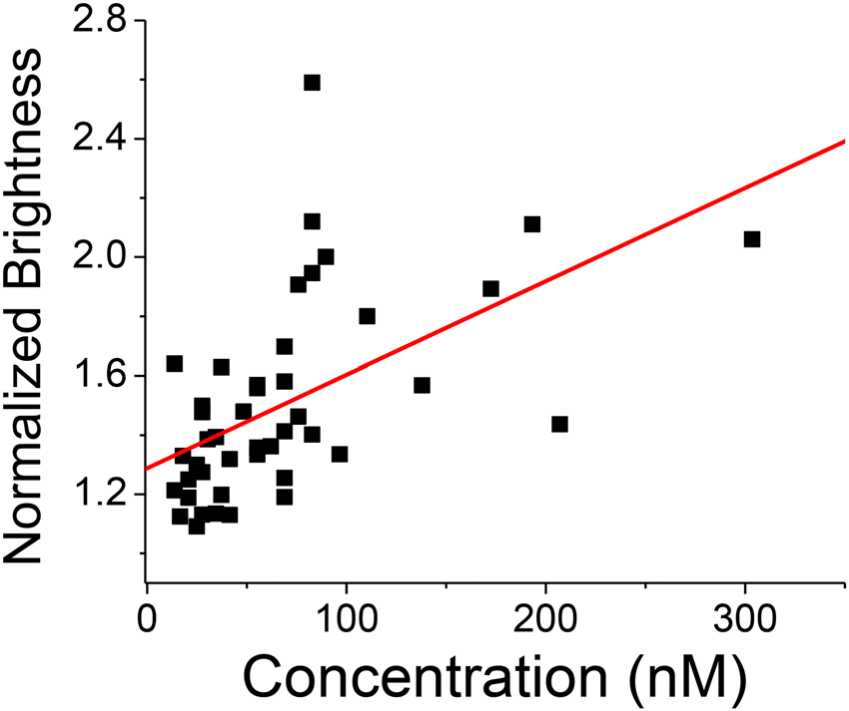
Arc-mCherry self-association is concentration dependent on the plasma membrane. TIRF N & B was used to obtain the average brightness of Arc-mCherry, which was converted to oligomeric state (normalized brightness) based on an mCherry standard (in solution and transfected in cells). The oligomeric state of Arc-mCherry on the plasma membrane was positively correlated with protein concentration (R^2^ = 0.45).

**Figure 5:**
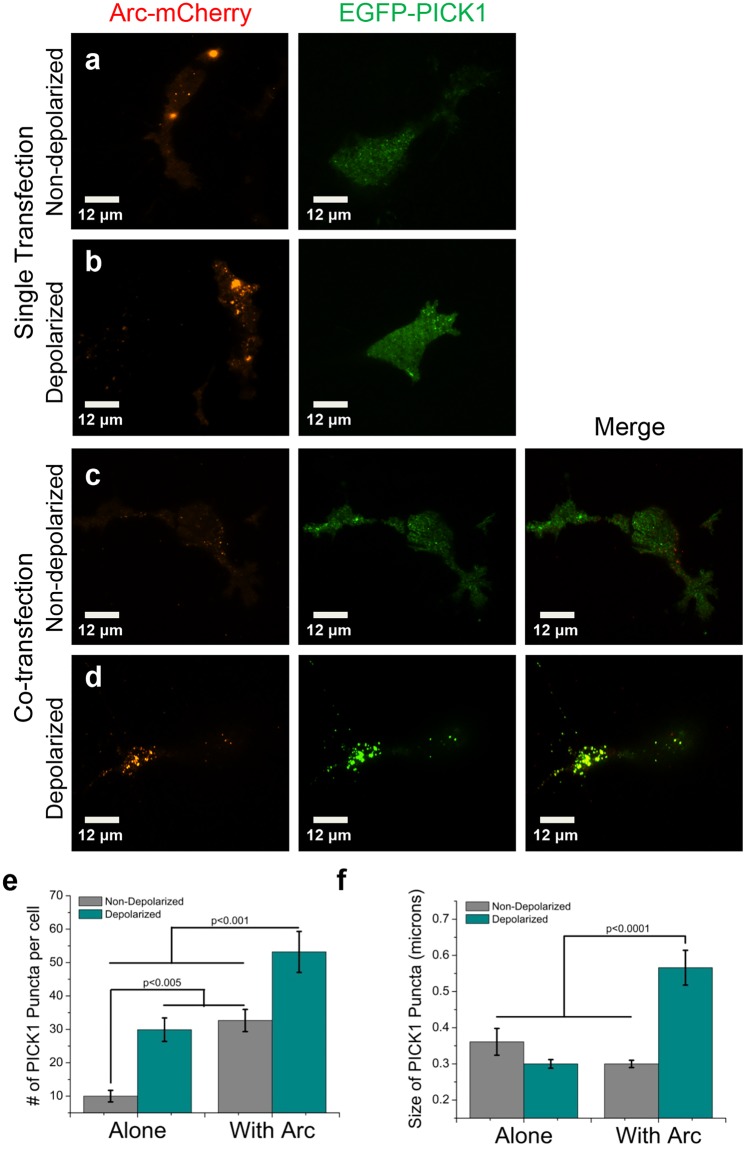
EGFP-PICK1 spatial organization is altered on the plasma membrane when co-transfected with Arc-mCherry and/or under depolarization conditions. TIRF intensity images of SH-SY5Y cells singly transfected with either Arc-mCherry or EGFP-PICK1 (**a**,**b**), as well as co-transfected cells (**c**,**d**) in either non-depolarizing buffer (**a**,**c**) or depolarizing buffer (**b**,**d**) reveal changes to the distribution of PICK1 resulting in a larger presence of puncta under co-transfected and depolarizing conditions. (**e**) Quantification of the number of apparent puncta formation in each condition shows significantly higher amounts of puncta are present due to co-transfection with Arc-mCherry as well as cellular depolarization. (**f**) Conversely, the average size of these puncta is only significantly affected by depolarization in the presence of Arc. (Statistical analysis was performed using a 2-way ANOVA with a Bonferroni post-hoc at an alpha level of p = 0.05. Error bars represent the SEM. n = 10 cells for each condition).

Both Arc and PICK1 are associated with the endocytosis of AMPARs and are therefore likely to be active at the plasma membrane. We used total internal reflection fluorescence (TIRF) microscopy combined with number and brightness (N&B) analysis to quantify alterations in the self-association state of Arc and PICK1 at the plasma membrane. We observed that the self-association of Arc-mCherry was concentration dependent and transitioned from monomer to dimer at higher concentrations (Fig. 4). Even though EGFP-PICK1 oligomerization was not concentration dependent (Supp. Fig. 3), we determined the average brightness value was consistent with a dimer (Table 3) which agrees with previous studies of PICK1 structure and function^25^. Interestingly, there was no significant change in the membrane self-association state of Arc-mCherry or EGFP-PICK1 when co-transfected (Table 3). However, we were able to observe a visual difference in the presence of puncta, altered spatial organization, and co-localization in activity induced cells. Under depolarizing conditions, co-transfected cells showed higher EGFP-PICK1 intensity and an increase in amount and size of PICK1 puncta compared to EGFP-PICK1 alone (Fig. 5). Specifically, the number of PICK1 puncta per cell is significantly increased by co-transfection with Arc-mCherry and further increased under depolarizing conditions (Fig. 5e). The average size of these puncta was only increased in the presence of Arc-mCherry and depolarizing buffer (Fig. 5f). Additionally, these EGFP-PICK1 puncta were found to co-localize significantly more with Arc-mCherry when depolarized (Pearson’s Correlation: 0.53 ± 0.05 vs. 0.77 ± 0.03 non-depolarized vs. depolarized, p < 0.05). These experiments provide substantial evidence that the interaction between Arc and PICK1 is present in the cytosol and at the plasma membrane. Furthermore, it is regulated by cellular depolarization and causes drastic changes in spatial dynamics.

**Table 3:** TIRF N&B analysis of membrane Arc-mCherry and EGFP-PICK1.

Transfected Protein^a^	Ave. Brightness (cpsm)	Norm. Brightness^b^
EGFP	0.10 ± 0.01	1.0
EGFP-PICK1	0.25 ± 0.05	2.5
EGFP-PICK1 + mCherry	0.22 ± 0.04	2.2
EGFP-PICK1 + Arc-mCherry	0.22 ± 0.05	2.2
mCherry	0.10 ± 0.02	1.0
Arc-mCherry	0.17 ± 0.03	1.7
Arc-mCherry + EGFP	0.17 ± 0.04	1.7
Arc-mCherry + EGFP-PICK1	0.16 ± 0.03	1.6

^a^For each condition, n > 40.

^b^Values were generated by dividing by monomer controls to each corresponding sample. We note for Arc-mCherry that there was a trend towards dimer at higher concentration and that this values represents the average self-association for the concentration range tested.

### The Arc binding region is located in the first half of the PICK1 BAR domain

**Figure 6:**
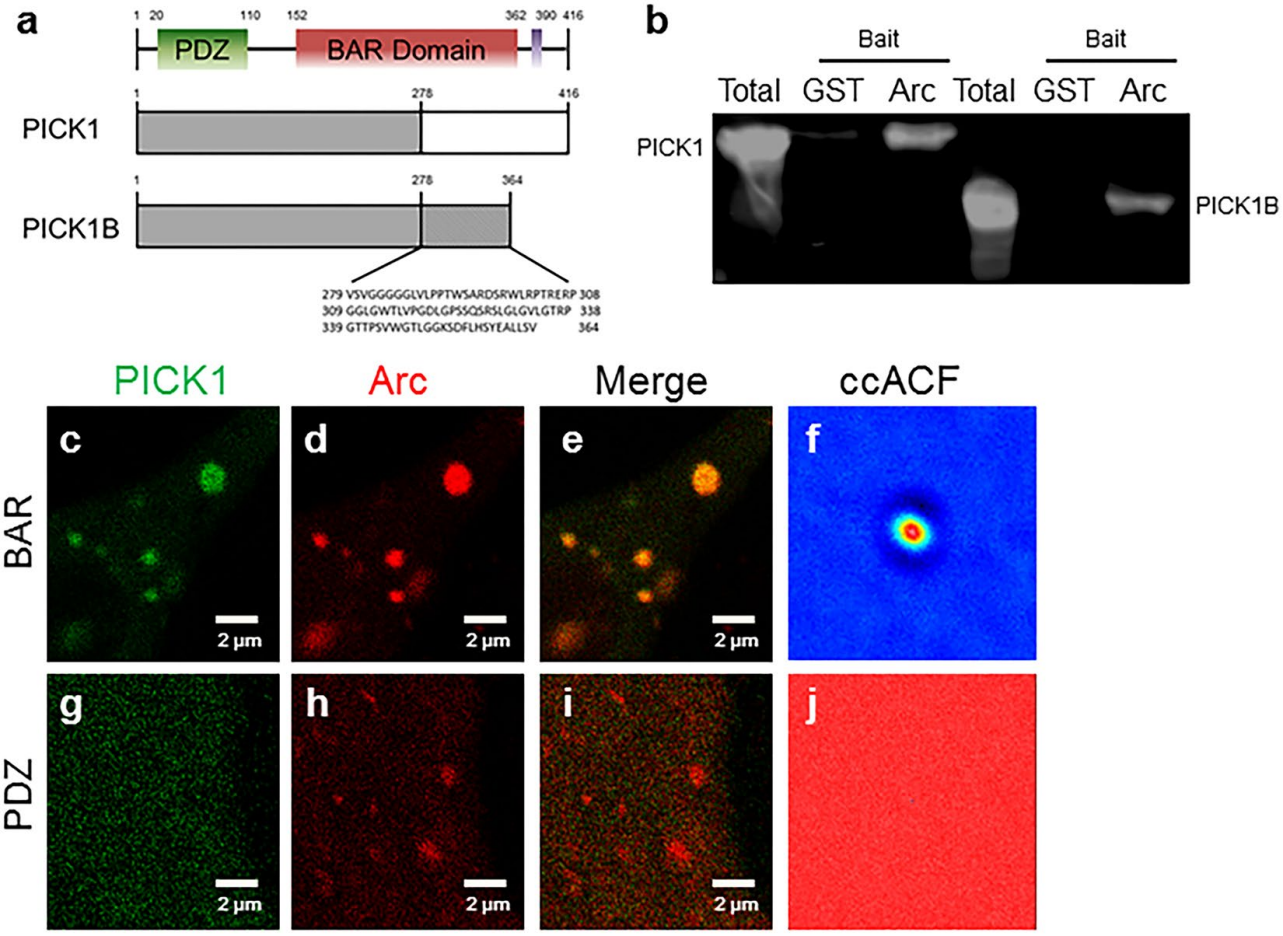
The Arc binding site is located in the PICK1 BAR domain. **(a)** A schematic representation of full length PICK1 and PICK1B highlights the structural domains of the protein (PDZ in green; BAR domain in red; and an acidic region in purple). PICK1B consists of the first 278 amino acids and the last 86 amino acids at the C-terminal end resulting in a truncated protein that is 52 amino acids shorter than the original sequence. (**b**) HEK293 cells were transfected with PICK1 (lane 1–3, positive control) or PICK1B (lanes 4–6). The lysates were loaded (lane 1 and 4, full lysate as a second positive control) or were subjected to a pull-down with purified GST-Arc (lane 3 and 6) or purified GST alone (lane 2 and 4, negative control), followed by Westernblot (WB) blotted for PICK1. (**c**–**e**) Representative intensity images of co-transfected SH-SY5Y cells containing Arc-mCherry and EGFP-PICK1-BAR or **(g–i)** EGFP-PICK1-PDZ under depolarizing conditions indicate that Arc-mCherry can associate with the PICK1-BAR domain but not the PICK1-PDZ. (**f**,**j**) Cross-correlation of the Arc-mCherry and EGFP-PICK1 fluctuation signals during RICS analysis produces **(f)** a characteristic autocorrelation function (ACF) indicating protein-protein interaction for cells transfected with the PICK1-BAR construct (**j**) while the PICK1-PDZ domain showed little to no correlation with Arc-mCherry. n = 15 cells for each condition. (The red to light blue distribution indicates the intensity of pixels that have correlated diffusion in both channels. Broader histograms (more red) indicate less correlation of the signals).

The experiments thus far have established that Arc and PICK1 have a direct interaction that occurs in the cytosol and at the plasma membrane and is stimulated under depolarizing conditions. Using a similar pull-down approach, we next sought to identify the potential binding region between Arc and PICK1. We utilized GST-Arc and a short form of PICK1 called PICK1B. The PICK1B construct contains the first 278 residues of the authentic PICK1 sequence and 85 C-terminal residues encoded by the intron sequence originating from a splicing skip (Fig. 6a). This truncation eliminates the third helix of the PICK1 BAR domain and the acidic domain involved in Arp2/3 interaction^26^. FLAG-PICK1B was transfected into HEK293 cells and was incubated with GST or GST-Arc which were loaded on to glutathione resin. WB analysis confirmed that GST-Arc, but not GST alone, was able to pull-down PICK1B from cell lysate (Fig. 6b). This indicates that the interaction between Arc and PICK1 is likely in the PDZ or the first half of the BAR domain.

To further determine which domain is the likely binding site for Arc, we used ccRICS analysis as conducted previously. SH-SY5Y cells were co-transfected with Arc-mCherry and either EGFP-PICK1-PDZ (residues 1–125) or EGFP-PICK1-BAR (residues 140–416). Intensity images of cells in depolarizing buffer suggest that Arc-mCherry co-localizes with the PICK1 BAR domain and not with the PDZ domain (Fig. 6c–e,g–i). Quantification of the fluctuation signals gave a characteristic ccACF (distribution from red to light blue) when Arc-mCherry was co-transfected with EGFP-PICK1-BAR which demonstrates that these two constructs are moving together throughout the cell (Fig. 6f). Conversely, no correlation was found when co-transfecting with EGFP-PICK1-PDZ (Fig. 6j). These experiments provide very strong evidence that Arc binds directly to PICK1 at the BAR domain between residues 152–278.

## Discussion

We used biochemical and biophysical analysis to establish a novel molecular connection between Arc and PICK1, two proteins known to facilitate the endocytosis of AMPARs during LTD^7,8,10^. Several laboratories have discovered interactions between Arc and endocytic proteins, such as dynamins and endophilin^9,10^. The established interaction of PICK1 with subunits of AMPARs and its function in AMPAR internalization makes it a good candidate to function as a scaffolding protein with Arc to direct endocytic proteins to AMPARs following neural activity. Our GST-Arc pull-downs from brain homogenates and with purified proteins demonstrate a direct interaction with PICK1. Until recently, it has been unknown how Arc directs specific internalization of AMPARs following rapid translation. Two labs have provided evidence of Arc interaction with TARPγ2 and AP-2 to explain this directed targeting^15,27^. Our data provide additional molecular knowledge to our rapidly growing understanding of the mechanism of Arc-regulated AMPAR association with the addition of PICK1.

Furthermore, through FRET studies of SH-SY5Y cells co-transfected with Arc-mCherry and EGFP-PICK1, we found that Arc interaction with PICK1 is enhanced under depolarizing conditions. This supports a role of the interaction in controlling activity-induced internalization of AMPARs. In a functional neuronal system, Arc expression levels are translationally regulated, with new Arc being translated in dendritic spines within 15 seconds of stimulation^28^. However, in our transfected SH-SY5Y system the increased interaction due to depolarization suggests that there are additional signals that regulate Arc and PICK1 association beyond elevated Arc concentration. Interestingly, FRET was primarily observed around the border of the cells indicating that this regulatory factor is localized near the membrane and following depolarizing activity. The most drastic shift in interaction was observed on the plasma membrane during depolarization. The homogenous distribution of PICK1 and Arc is considerably altered due to puncta structures that contain both Arc and PICK1 when co-transfected and depolarized. Therefore, the interaction of Arc and PICK1 within neuronal cells may not be exclusive to having both proteins present, even at high concentrations, but rather require posttranslational modifications or molecular interactions^29^. Additional studies need to be conducted to evaluate the effect of Arc interaction with PICK1 on the surface concentration and localization of AMPARs.

Due to the use of transfection to introduce our DNA construct into SH-SY5Y cells, there can be a large range of expression cell to cell. Using N&B analysis and TIRF microscopy, we determined the average oligomerization state of our constructs. Though Arc oligomerization has been demonstrated by several methods using purified protein^9,30^, it has not previously been shown if Arc self-associates in neurons. Here we show, to the first of our knowledge, evidence that Arc-mCherry forms dimers and higher-order oligomers in SH-SY5Y cells. Additionally, we observed a concentration dependence of Arc-mCherry self-association on the plasma membrane which may contribute to the function of Arc. Due to similarities between the structure of Arc fragments and Gag capsid proteins, it has been speculated that Arc may be primarily active in the dimeric form^31,32^. We hypothesize that the localized, rapid expression of Arc in response to neural activity may adequately increase the microconcentration of Arc and lead to an increased propensity to self-associate. This may serve as an additional mechanism to control the spatial and temporal activity of Arc. Additional studies need to be conducted to evaluate the effect of Arc interaction with PICK1 on the surface concentration and localization of AMPARs. Future studies should be conducted in a true neuronal system to establish Arc oligomerization and study its effects on Arc function.

There are two functional domains of PICK1 that would likely serve as the binding site for Arc, the PDZ domain and the BAR domain. Using pull-downs and ccRICS, we show evidence that Arc interaction likely occurs at the BAR domain of PICK1. Heterodimerization of PICK1 with another BAR domain-containing protein, ICA69 (islet cell autoantigen of 69 kDa), was previously reported to inhibit PICK1 homodimerization^33,34^. It is also is valuable to note that Arc was found to bind to the third helix of the endophilin 3 BAR domain^10^. Therefore, it is plausible that Arc might have specificity towards BAR domain containing proteins.

**Figure 7:**
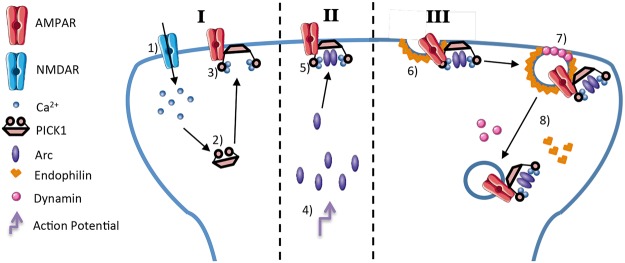
A proposed model depicts the control of AMPAR surface expression through Arc/PICK1 interaction. Our proposed model suggests a potential mechanism through which Arc is selectively targeted to AMPARs during LTD. Phase I: (1) Ca2+ enters through activated NMDA receptors (2) and binds to PICK1 (3) leading to an electrochemical conformational change allowing PICK1 to preferentially bind to AMPARs. Phase II: (4) A depolarizing event increases the local expression and concentration of Arc protein in the post-synaptic terminal (5) which leads to increased binding to activated PICK1. Phase III: (6) Arc recruits endophilin and (7) dynamin causing membrane curvature (8) and the endocytosis of AMPARs from the plasma membrane.

Overall, our studies help to answer the question of how Arc directly targets the internalization of AMPARs. We propose a model by which (1) sufficient depolarization leads to calcium entry through NMDA receptors. (2) Elevated calcium levels lead to conformation changes in PICK1 that (3) increase its association with AMPARS^16^. (4) Further synaptic stimulation leads to rapid expression of Arc and (5) allows for interaction with PICK1. (6) Arc brings dynamin and endophilin to the membrane and (7) increases polymerization of dynamin at the neck of the AMPAR containing vesicles (8) leading to increased AMPAR internalization (Fig. 7). Our understanding of the molecular actions that underlie learning and memory are rapidly increasing. Though additional studies are needed to fully understand the function of Arc-PICK1 interaction, our data help to connect the gap between depolarizing events and AMPAR regulation.

## Materials and Methods

### Preparation of crude synaptosome extract

Mouse brains were homogenized in buffer (0.32 M sucrose, 4 mM HEPES, pH 7.5, 1 mM EDTA, 1 mM EGTA, 0.2 mM PMSF, and protease inhibitor cocktail). Homogenate was centrifuged for 15 min at 800 × g, and the supernatant was then centrifuged for 50 min at 17,000 × g. The pellet contains crude synaptosome, P2 fraction. P2 pellet was resuspended in resuspension buffer (50 mM HEPES, pH 7.5, 0.1 M NaCl, 0.1% Triton X100, 10% glycerol, 1.5 mM MgCl_2_, 0.2 mM PMSF, and protease inhibitor cocktail) and centrifuged for 30 min at 100,000 × g. The supernatant was used for analysis. Mouse brains were harvested in accordance with protocols approved by UT Southwestern (IACUC: A3472-01; K. Huber).

### Expression and Purification of GST-Arc and His-PICK1

Mouse Arc cDNA was cloned into pGST. parallel 1 vector and purified as previously described^15^. Human PICK1 was introduced into the pQE-80L expression vector and transformed into BL21-Codon(+) DE3 (Agilent Technologies). Bacterial cultures were incubated at 37 °C in LB containing ampicillin (100 mg/mL). At OD_600_ = 0.9, cells were cooled to 18 °C and induced with 0.5 mM IPTG, cultured at 18 °C for 20 h and centrifuged at 4000 × g for 40 min. The pellet was resuspended in lysis buffer (10 mM HEPES, pH 8, 150 mM NaCl, 1 mM PMSF, rLysozyme, benzonase nuclease, 1X fastbreak and 10% glycerol) and lysed by one freeze thaw cycle and sonification. The cells were harvested by centrifugation at 7,000 RPMs and 4 °C for 40 min and dialyzed overnight in loading buffer (10 mM HEPES, pH 8, 150 mM NaCl, 30 mM imidazole, 1 mM PMSF and 10% glycerol). The solution was then loaded onto a 5 mL HisTrap column (GE Healthcare) at 3.0 mL/min and washed with washing buffer (10 mM HEPES, pH 8, 150 mM NaCl, 80 mM imidazole, 1 mM PMSF and 10% glycerol). His-PICK1 was eluted from the column with elution buffer (10 mM HEPES, pH 7.5, 150 mM NaCl) and 1 mL fractions were collected off the column. GST-Arc was similarly purified using glutathione agarose resin (Pierce) and eluted with 50 mM L-glutathione reduced. The protein purity was assessed using SDS-PAGE and Westernblot analysis using a polyclonal anti-PICK1 antibody (N-18, Santa Cruz Biotechnology) or anti-GST antibody (27-4577-01, GE Healthcare). Fluorescently labeled secondary antibodies for Infrared Imaging System were from LI-COR. Protein was exchanged into working buffer (10 mM HEPES, pH 7.5, 150 mM NaCl, 1 mM DTT and 1 mM PMSF) by dialysis. When needed, protein was further purified by running the sample over a SEC (Sephacril S200, GE Healthcare). Both Arc and His-PICK1 were centrifuged at 214,000 × g for 15 min at 4 °C prior to all assays to remove potential aggregates.

### Cell culture

HEK293 cells and SH-SY5Y cells (ATCC) were cultured in DMEM F12 containing 15 mM HEPES, 1.5 mM sodium pyruvate, 1x Antibiotic-Antimycotic and 10% FBS in tissue culture-treated T75 flasks at 37 °C and 5% CO_2_. All tissue culture reagents were from Life Technologies (Carlsbad, CA, USA) unless specified. Cells were lifted with a 0.08% solution of trypsin-EDTA in PBS. For imaging, cells were plated onto uncoated 10 mm glass bottom dishes (MatTek) and transfected with Lipofectamine 3000 (Invitrogen) following the manufacturer’s protocol with 0.2–1.5 μg DNA plasmid per dish. A mouse variant of Arc was ligated to mCherry at the C terminus. A human variant of PICK1, PICK1 PDZ domain, and PICK1 BAR domain were ligated to EGFP at the N terminus. For depolarization studies, cells were washed in PBS and imaged in either depolarizing buffer (42 mM NaCl, 100 mM KCl, 0.6 mM MgSO_4_●7H_2_O, 2.5 mM CaCl_2_, 6 mM D-glucose, 10 mM HEPES, pH 7.4) or nondepolarizing buffer (135 mM NaCl, 5 mM KCl, 0.6 mM MgSO_4_●7H_2_O, 2.5 mM CaCl_2_, 6 mM D-glucose, 10 mM HEPES, pH 7.4) following 15 min incubation.

### Pull-down of PICK1 from cell extracts

HEK293 cells were transfected with FLAG-PICK1 (either full length PICK1 or PICK1B), and lysed (50 mM Hepes, pH 7.5, 0.1 M NaCl, 0.1% Triton X100, 10% glycerol, 1.5 mM MgCl_2_, 0.2 mM PMSF, and protease inhibitor cocktail). Lysates were centrifuged at 20,000 × g for 15 min and the supernatants were incubated with GST-Arc or GST (negative control) coupled to glutathione resin. After a 1 h incubation with constant rotation, the samples were centrifuged (2 min at 1,000 × g), washed 3 times with lysis solution, then the bound proteins were eluted from the resin with 50 mM glutathione. The eluates were subjected to immunoblotting with a monoclonal anti-Flag antibody (Sigma).

### Pull-down of PICK1 from recombinant expression

Soluble His-PICK1 (1, 5 and 10 µM) was mixed with GST-Arc (1 µM), GST (1 µM) bound to glutathione-Agarose, or glutathione-agarose resin alone in buffer (20 mM HEPES, pH 7.5, 100 mM NaCl, 1 mM DTT and 1 mM PMSF for 1 h at 4 °C). After 1 h incubation with constant rotation, the mixtures were centrifuged at 700 × g for 3 min, washed 3 times, and the supernatants, unbound and eluted, were collected. PICK1/GST complexes bound to glutathione-Agarose beads (150 µL) were collected by incubation with 50 mM glutathione. Analysis of bound and free PICK1 were determined using SDS-PAGE and BCA assay.

### Two-photon confocal imaging

Fluctuation measurements were recorded on an Alba fluorescence correlation spectrometer (ISS, Champaign, IL), equipped with x-y scanning mirror, connected to a Nikon TE2000-U inverted microscope (Nikon, Melville, NY) with a PlanApo VC 60 × 1.2 NA water objective. Two-photon excitation of EGFP-PICK1 and Arc-mCherry was provided by a Chameleon Ultra (Coherent, Santa Clara, CA) tuned to 1,000 nm. Fluorescence emission was spectrally filtered through a 680 nm short-pass filter (FF01-680; Semrock, Rochester, NY) and dichroic mirror (700dcxru, Chroma, Bellows Falls, VT) with intensity of EGFP and mCherry collected on individual PMTs. Cells were imaged in a humidified enclosed chamber kept at 37 °C (Tokai Hit, Fujinomiya, Shizuoka, Japan). An objective heater wrapped round the neck of the objective was used to minimize temperature drifts (with a 20 min delay to equilibrate the temperature prior to imaging) and the collar of the objective was adjusted to compensate for temperature and thickness of the coverslip.

Raster imaging correlation spectroscopy (RICS) was used to examine the protein dynamics in live cells^11,35^. Briefly, 12.8 micron (50 nm pixels) regions of interest were selected from the fluorescence image. The pixel sampling time was 12.5 μs, each frame had 256 × 256 pixels, and each measurement lasted approximately 1 min (100 frames). The laser power at the sample was <1 mW. The beam waist (ω_0_) calibration was achieved by measuring the autocorrelation curve of fluorescein (~20 nM) in 0.01 M NaOH, and fitted with a diffusion rate of 430 µm^2^/sec, which were performed before each day’s measurement. The typical values of ω_0_ were at the range of 0.35–0.40 µm.

For the photon counting histogram (PCH) measurement, the laser excitation point was selected in a region in the cytosol away from the nucleus and plasma membrane. Intensity fluctuations were recorded for approximately 4 min with sampling rate of 50,000 Hz. Monomer brightness of EGFP was obtained by averaging cells transfected with monomeric EGFP at various protein concentrations. Brightness values were calculated using Vinci software with incorporation of the dead-time of the detectors (50 ns).

Fluorescence lifetime imaging microscopy (FLIM) measurements were recorded through an ISS A320 FastFLIM box coupled to the Ti:Sapphire laser, which produces 80-fs pulses at a repetition rate of 80 MHz, and photo multiplier detector (H7422P-40, Hamamatsu, Hamamatsu City, Japan). EGFP was excited at 920 nm and the fluorescence signal was filtered away from excitation light through a 520 nm bandpass filter (FF01-520/35; Semrock Rochester, NY) mounted in front of the detector.

### TIRF imaging

Images were recorded on a Nikon Eclipse Ti Total Internal Reflection Fluorescence microscope using a 60 × 1.45 NA oil objective. A cascade 512B EMCCD camera (Photometrics, Tucson, Az) equipped with a dual view image splitter was used to image EGFP and mCherry at 100 frames/s. Both proteins were excited simultaneously at 488 nm for EGFP and 561 nm for mCherry using a triple band excitation filter (405/488/594 nm; Chroma, Bellows Falls, VT) within the infinity space.

### Data analysis

PCH was analyzed using VistaVision software (ISS). RICS, N&B, FRET, and puncta size were analyzed using Globals for Images (SimFCS software from the Laboratory for Fluorescence Dynamics) as previously described^11,35–38^. ImageJ/Fiji was used for TIRF co-localization analysis^39^. Comparison between groups was carried out using multivariate ANOVA using Bonferroni post-hoc. Statistical significance was accepted when p < 0.05. Statistical analyses were performed using SAS University software.

## Electronic supplementary material

Supplemental Figure

## Data Availability

The authors will make available all data and materials. Interested parties should contact N.G.J. (njames4@hawaii.edu) to make requests.
