# Supplementary material for: Arc/Arg3.1 has an activity-regulated interaction with PICK1 that results in altered spatial dynamics
Source: Sci Rep. 2018 Oct 2;8:14675. doi: 10.1038/s41598-018-32821-4 (PMC6168463; doi:10.1038/s41598-018-32821-4)

Arc/Arg3.1 has an activity-regulated interaction with PICK1 that results in altered spatial dynamics

Brandee M. S. S. Goo[1], Bethany J. Sanstrum[1], Diana Z. Y. Holden[1], Yi Yu[1], and Nicholas G. James[1*]

Supplemental Figure 1: GST-Arc pull-down of PICK1 from Brains. GST-Arc co-immunoprecipitates with PICK1 while our negative control, GST, was not found to interact. Brain lysates were subjected to pulldown with GST-Arc followed by Westernblot (WB) with anti-PICK1 antibody. White spaces have been placed between each lane to indicate lanes were not run adjacent but rather place together for comparison purposes.

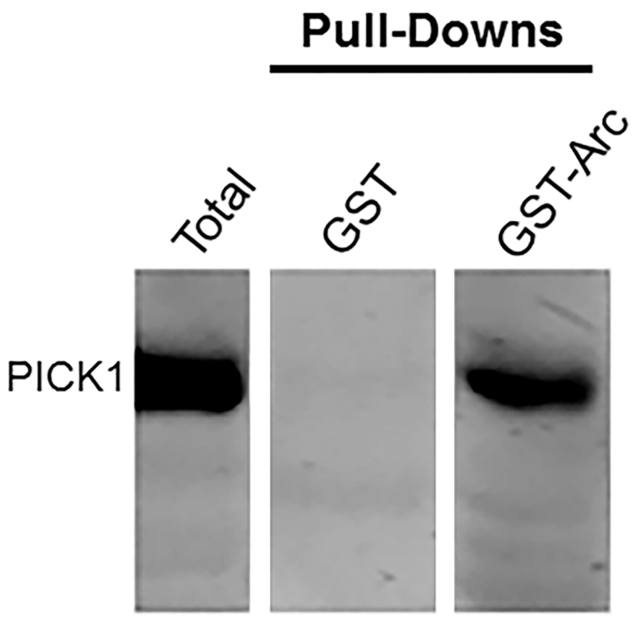

Supplemental Figure 2: GST-Arc pull-down of PICK1 from Synaptosomes (Fig. 1A from Manuscript). Lane 1,2,10 and 17: Total amount of PICK1; Lanes 3,4,11, and 12: PICK1 pulled-down using GST-Arc; Lanes 5,6,13 and 14: Total amount PICK1 pulled down using GST; Lanes 7,8,15, and 16: PICK1 pulled down using GST-Grb2; Lane 9: LI-COR MW ladder (150 and 50 kDa highlighted). Lanes 2, 3, 6 and 8 were used for image 1A in Manuscript. These lanes have been marked with black spacer bars to direct the reader to regions that were removed from the final manuscript figure.

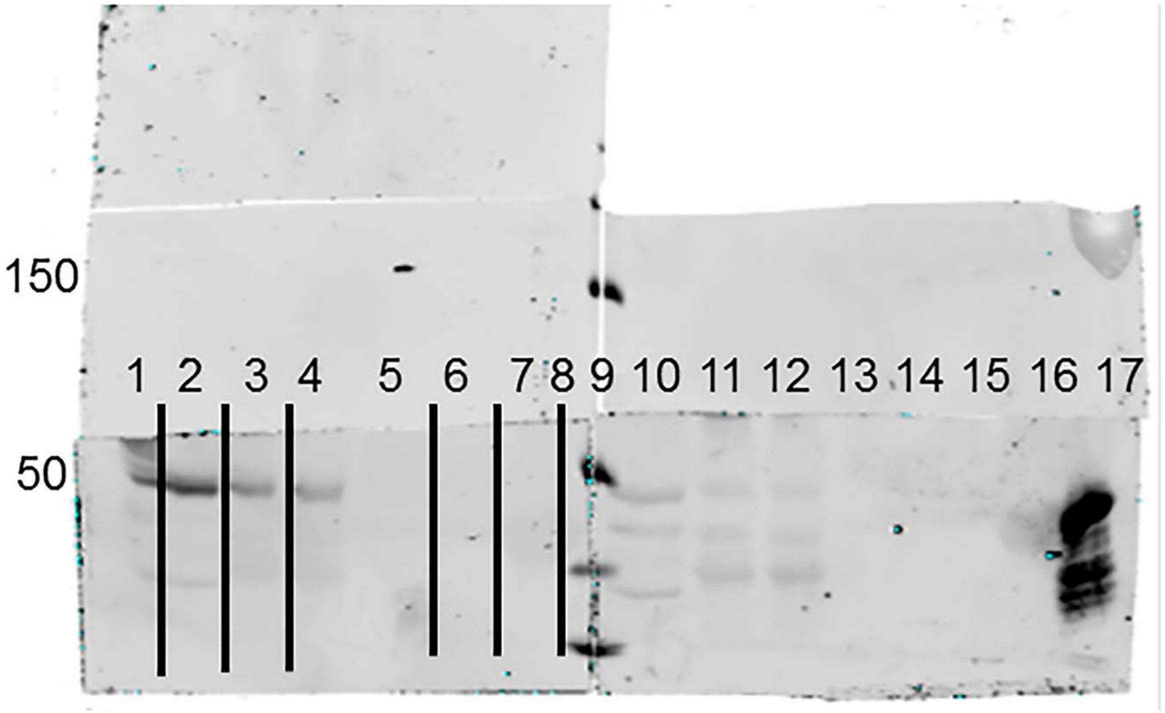

Supplemental Figure. 3: EGFP-PICK1 self-association is not concentration dependent on the plasma membrane. TIRF N&B was used to obtain the average brightness of EGFP-mCherry, which was converted to oligomeric state (normalized brightness) based on an EGFP standard (in solution and transfected in cells). The oligomeric state of EGFP-PICK1 on the plasma membrane did not show a trend ($R^2 < 0.01$).

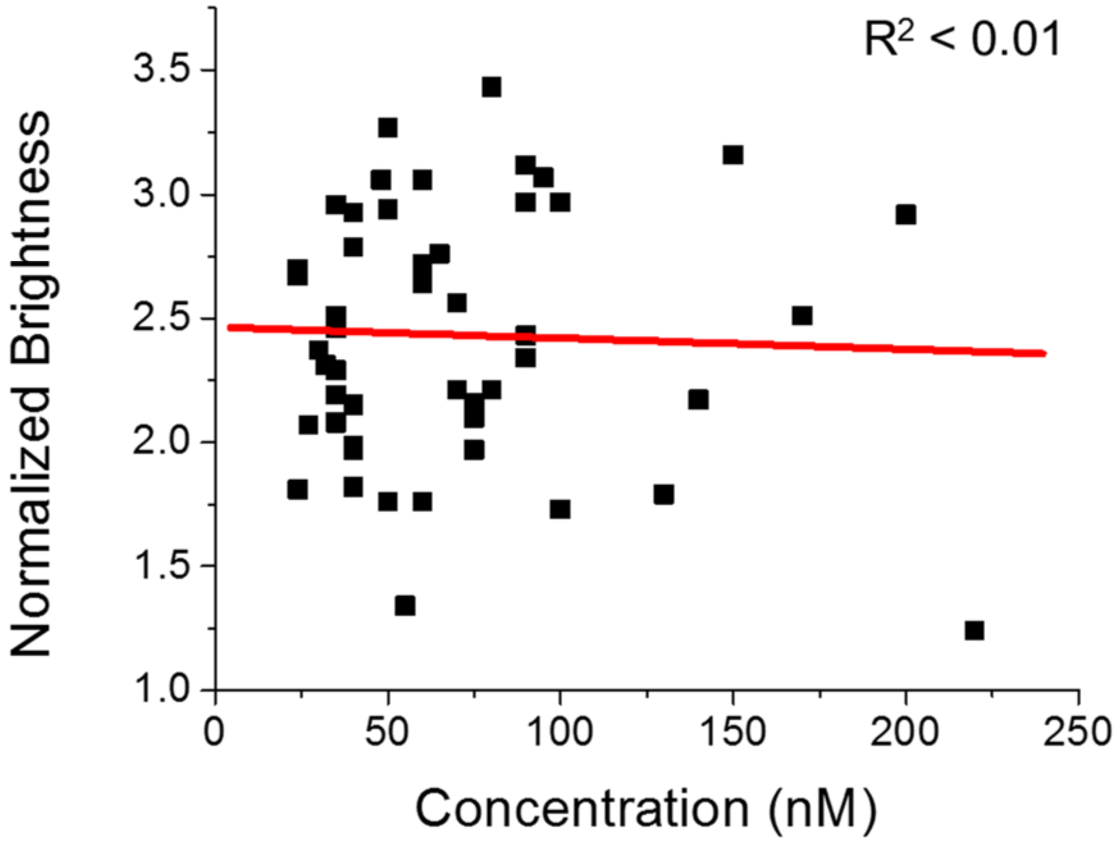

Supplemental Figure 4: GST-Arc pull-down of PICK1B (Fig. 6B in Manuscript). Lane 1: LI-COR MW ladder, Lanes 2 and 9: Total amount of PICK1 from transfected HEK293 cells; Lanes 3 and 10: PICK1 pulled down using GST; Lanes 4 and 11: PICK1 pulled-down using GST-Arc; Lanes 5 and 12: Total amount of PICK1B from transfected HEK293 cells; Lanes 6 and 13: PICK1B pulled down using GST; Lanes 7 and 14: PICK1 pulled down using GST-Arc. Green: PICK1 and PICK1B; Red: GST and GST-Arc. White box denotes the cropped region presented in Figure 6B.

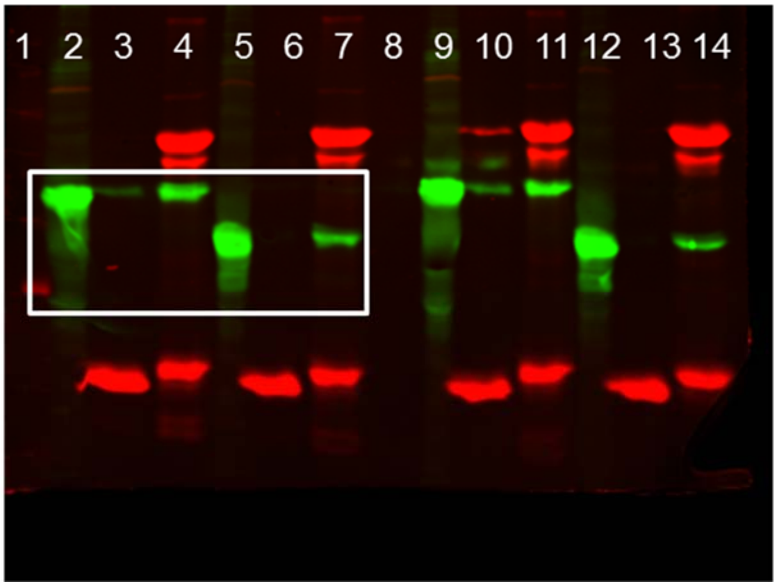

Supplement: Supplementary file 1 — Supplemental Figure [file 41598_2018_32821_MOESM1_ESM.pdf]
